# Pyridoxamine is a substrate of the energy-coupling factor transporter HmpT

**DOI:** 10.1038/celldisc.2015.14

**Published:** 2015-07-14

**Authors:** Tingliang Wang, Armando Jerome de Jesus, Yigong Shi, Hang Yin

**Affiliations:** 1 Ministry of Education Key Laboratory of Protein Science, Tsinghua University, Beijing, China; 2 Tsinghua-Peking Joint Center for Life Sciences, Center for Structural Biology, School of Life Sciences and School of Medicine, Tsinghua University, Beijing, China; 3 Department of Chemistry & Biochemistry, the BioFrontiers Institute, University of Colorado Boulder, Boulder, CO, USA; 4 Center of Basic Molecular Science, Department of Chemistry, Tsinghua University, Beijing, China

**Keywords:** ECF transporters, S-component, HmpT, molecular dynamics, mass spectrometry, gating mechanism

## Abstract

Energy-coupling factor (ECF) transporters belong to a novel family of proteins that forms a subset within the ATP-binding cassette (ABC) transporter family. These proteins are responsible for the uptake of micronutrients in bacteria. ECF transporters are composed of four proteins: the A- and A′-components, the T-component and the S-component. One of the ECF transporters, named HmpT, was crystallized in the *apo* form with all four components. It is currently unknown whether HmpT serves as a transporter for hydroxymethyl pyrimidine or the different forms of vitamin B6 (pyridoxine, pyridoxal or pyridoxamine). Using a combination of molecular dynamics (MD) simulations and mass spectrometry, we have identified pyridoxamine to be the preferred substrate of HmpT. Mass spectra show that the mass of the substrate from the HmpT–substrate complex matches that of pyridoxamine. MD simulations likewise indicate that pyridoxamine interacts most strongly with most of the conserved residues of the S-component (Glu 41, His 84 and Gln 43) compared with the other vitamin B6 forms. Furthermore, the simulations have implied that loops 1 and 5 of the S-component can participate in the gating action for HmpT.

## Introduction

As early as the late 1970s, proteins that transport B-vitamins into bacteria have been observed [1–3], but it was only recently that these proteins, designated as energy-coupling factor (ECF) transporters, have been identified to form a novel subset within the ATP-binding cassette (ABC) transporter family [4–9]. The first detailed structure to be solved did not come until 2010 when Zhang *et al.* reported the solved structure of a component of a riboflavin transporter [10], and it was not until 2013 when the crystal structures of the whole transporter were published [11, 12]. These transporters are responsible for the uptake of micronutrients, such as vitamins, metals or amino acids, in bacteria and archaea [11–15]. As these organisms would often lack the capacity to synthesize these transported micronutrients [6, 16, 17], ECF transporters are essential to survival, and thus, present attractive targets for potential antibiotic drugs.

**Figure 1:**
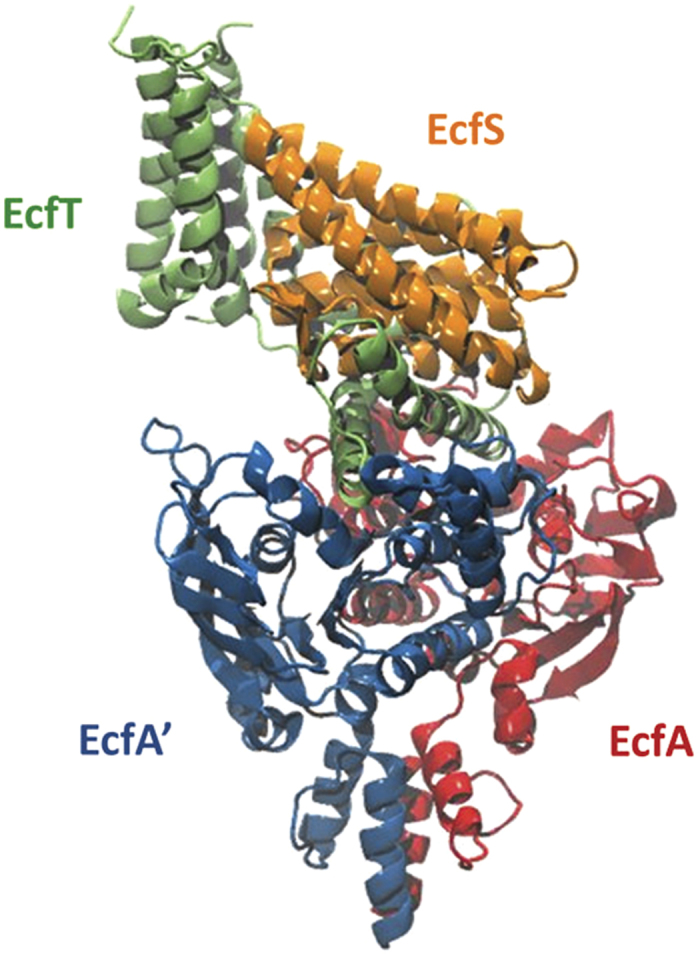
Cartoon representation of the ECF transporter HmpT. The regions in blue and red are the A-components while the S- and T-components are colored orange and green, respectively.

**Figure 2:**
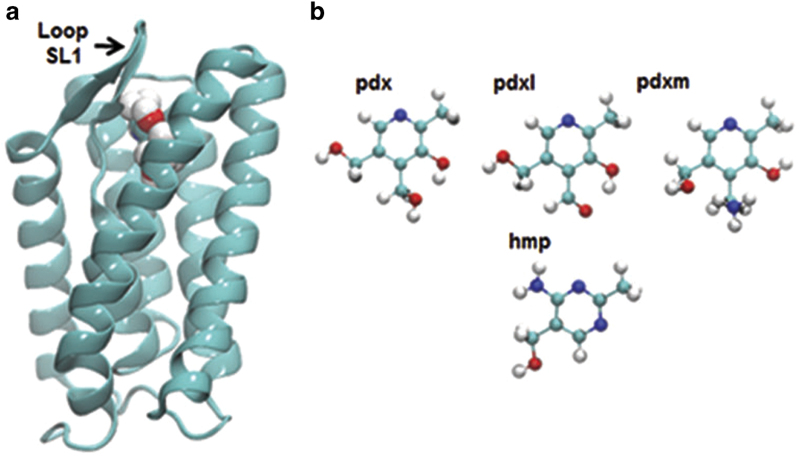
Structure of the S-component, HmpT and substrates used in the simulation. (**a**) Features a snapshot of pyridoxamine inside the binding pocket of the S-component, HmpT (4HZU, [11]), that is being simulated in the open orientation. In this snapshot, loop 1 is starting to close over the opening of the binding pocket. (**b**) Displays the structures of the ligands that were simulated with HmpT: pyridoxine (pdx), pyridoxal (pdxl), pyridoxamine (pdxm) and hydroxymethyl pyrimidine (hmp). The white, cyan, blue and red balls represent hydrogen, carbon, nitrogen and oxygen atoms, respectively.

Currently, there are six known structures of these proteins that transport folate (FolT [12]), riboflavin (RibU [10]), thiamine (ThiT [18]), biotin (BioY [13]), nickel (NikM [15]) and pyridoxine (PdxU2 [11]). Figure 1 displays a cartoon representation of the structure of the whole ECF transporter reported by Wang *et al.* PdxU2 (Figure 2, pdx) was initially named HmpT [11] as it was thought to transport hydroxymethyl pyrimidine, but genomic context analysis also suggests that it transports pyridoxine [7]. Thus, currently, the substrate for PdxU2 is unknown. As pyridoxine is only one of the active forms of vitamin B6, it is also unknown whether PdxU2 can transport other active forms (pyridoxal and pyridoxamine, Figure 2b, pdxl and pdxm) of this vitamin. A combination of computational and experimental approaches will be employed to identify the possible substrate of HmpT and to suggest a potential mechanism for how the substrate interacts with the binding pocket and how the binding pocket is gated.

ECF transporters have two main modules: an S-component (EcfS) that binds the substrate and a larger ECF module [7, 19–21]. The ECF module is composed of three components: the T-component (EcfT) and two similar cytosolic ATPases known as the A-components (EcfA and EcfAʹ). These ATPases (also known as nucleotide-binding domains) are a hallmark of the ABC family of transporters that use these domains to couple ATP hydrolysis to the transport of molecules across the lipid bilayer [7, 9, 22]. This coupling, in turn, is mediated by the EcfT, a transmembrane protein that links the A-component complex to EcfS [19, 20] and which can be identified by two short conserved Ala-Arg-Gly motifs [7]. The S-component presents a major structural difference between an ECF transporter and a canonical ABC transporter. Whereas the substrate-binding protein of ABC transporters is located in the periplasm or is tethered to the membrane of bacteria, EcfS is a membrane-embedded protein [11, 12]. Depending on whether the ECF module is unique to an S-component or not, ECF transporters are typically classified as either a group I or a group II transporter [7, 19, 20]. For group I transporters, the ECF module and the S-component are encoded in the same operon leading each EcfS protein to have a dedicated ECF module. In the case of group II transporters, the genes for the S-component are scattered in the genome and are not located in the same operon as the genes for the ECF modules. The S-components of this latter group share a single ECF module.

The S-component has a high affinity for substrates, typically in the nanomolar to sub-nanomolar range [5, 13, 23, 24]. This component confers substrate specificity to ECF transporters. There is a low degree of sequence similarity among the different EcfS proteins but the global structures remain conserved [6, 13, 18]. The six solved transporter structures mentioned beforehand all contain EcfS structures: two were crystallized in the *apo* form together with the ECF modules (the folate transporter FolT [12] and pyridoxine transporter PdxU2 [11, 19]) while the other four were crystallized in isolation from the ECF module. The latter four are RibU [10], ThiT [18], BioY [13] and NikM [15] that, respectively, transport riboflavin, thiamine, biotin and nickel. NikM, a metal transporter, is currently the only solved structure of an exclusively group-I EcfS protein [15, 19].

Common among EcfS proteins is the presence of six transmembrane helices (SH1-6) that are arranged in a cylindrical manner. For metal transporters of the group I classification, in particular NikM and CbiM, an additional transmembrane segment is present [4, 15]. The helices, SH1 and SH3-5, each contain around 20 residues. SH2 is the shortest with only 9–11 residues, while SH6 is the longest with around 40 residues. The longest loop, SL1, connects SH1 and SH2, while SH2 and SH3 is connected by the short loop SL2. The loops, SL3–5, meanwhile, respectively, connects the following helices: SH3-SH4, SH4-SH5 and SH5-SH6. The substrate binding site is surrounded by SH4-6 and loops SL1, SL3 and SL5 [20]. These loops might also have a role in substrate binding by acting as lids that close over the binding site [20, 25, 26]. ThiT, RibU, BioY and NikM (the substrate-bound S-components crystallized with no other components) were predicted to have the substrate binding site facing the extracellular side of the membrane with both the N- and C-terminals facing toward the cytoplasm [10, 13, 15, 18, 19]. However, the reported structures of the complexes of FolT and HmpT with the ECF module revealed a remarkable orientation for these S-components [11, 12]. These complexes show an inward-facing S-component that appears to have ‘toppled over’ [19] compared with the predicted orientation of the *apo* S-component structures. This orientation is consistent with the absence of a substrate in the crystal structure. In addition, the binding pockets for these inward-facing, *apo* S-components are widely open compared with those of RibU, ThiT and BioY [20]. The complete ECF structures show that the EcfS protein does not have direct contact with the A-components and interacts almost exclusively with EcfT protein [19, 20].

The EcfT protein or T-component is composed of eight helices (TH1-8) that adopt an L-shaped cleft on which the EcfS protein situates itself [11, 12]. The helices, TH1-4 and TH8, span the membrane and are connected by the horizontally placed TH5 to the cytoplasmic helices, TH6 and TH7. The latter two helices, in turn, couple with the A-components of the ECF transporter and, in a proposed mechanism, are hypothesized to have a role in transducing the motions of the A-components to the EcfS protein [11].

A very speculative mechanism proposed by Wang *et al*. [11] has been described as a ‘toppling mechanism’ [19], which is a variation of what is termed as the alternating access model. This model describes protein transport as a process wherein the extracellular and cytosolic sides of the membrane alternate access to the substrate-binding site. On the basis of vicinity of the substrate-binding site in the isolated S-components, it is suggested that substrate binding occurs near the extracellular side of the membrane with loops SL1, SL3 or SL5 acting as gates that control access to the binding site. On the basis of complete ECF structures crystallized, a substrate-bound EcfS protein is then proposed to topple over toward the orientation of the FolT or HmpT structures to deliver the substrate to the cytosol.

To identify its natural substrate, we herein studied the interaction of HmpT with hydroxymethyl pyrimidine and the different forms of vitamin B6. Knowledge of the substrate is significant not only for understanding micronutrient transport in bacteria but also in the search of possible targets for novel antibiotic pharmaceuticals. A substrate sample extracted from HmpT is subjected to mass spectrometry analysis for identification. For further exploration of the interaction of the identified substrate with the binding pocket, molecular dynamics (MD) simulation is employed to model HmpT in a membrane environment. HmpT is simulated in both the ‘closed’ and ‘open’ conformations. The closed conformation is taken directly from the *apo* structure of the HmpT with the S-component in the ‘toppled’ conformation [11]. The open conformation results from a rotation of the whole S-component such that principal axis of the component is relatively parallel to the *z*-axis. This also results in an orientation where the binding pocket opening faces the outside of the implicit bilayer. The structures of these two HmpT systems are compared to study whether the orientation of HmpT affects its behavior in the presence of ligands. Six residues in the binding pocket are conserved across a number of different S-components [11], and mutations of these residues are studied experimentally and computationally. Mutations of the ‘hot spot’ residues resulted to a significant drop in protein expression, which is consistent with the speculation that these residues contribute importantly to transport these essential nutrients [17, 26]. This combination of experimental and computational approaches, thus, identifies the substrate and provides microscopic-level and dynamic information on how the substrate interacts with HmpT.

## Results and Discussion

### Mass spectra identifies pyridoxamine as the substrate extracted from HmpT

One overarching question in studying the HmpT transporter remains to be the identification of its substrate due to the fact that the ECF structure of which HmpT is a part was crystallized in its *apo* form. Numbering among the possible substrates are pyridoxine and hydroxymethyl pyrimidine. Since pyridoxine is one of the active forms of vitamin B6, it leads to some speculation as to whether HmpT transports only pyridoxine or whether other forms of vitamin B6, such as pyridoxal and pyridoxamine, can also act as the substrate.

**Figure 3:**
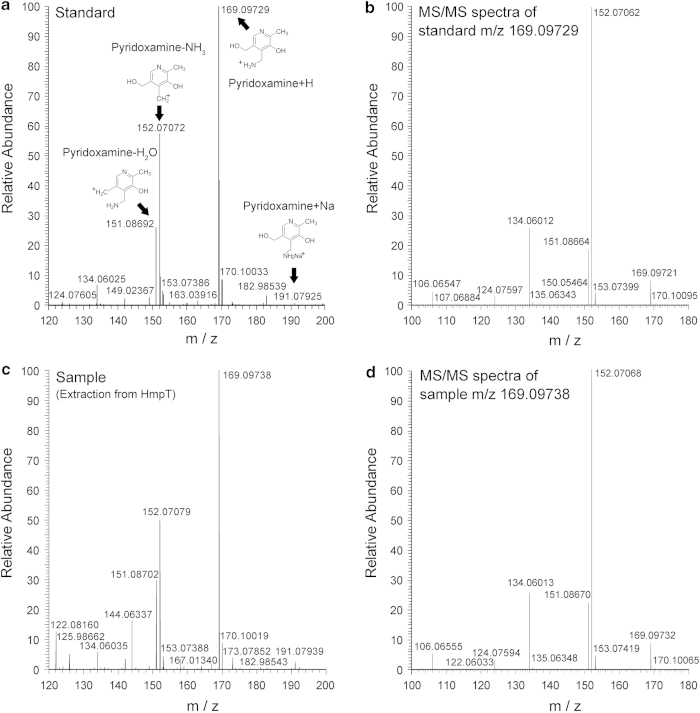
Mass spectra of the standard and the substrate extracted from EcfS protein. (**a**, **b**) Show the pyridoxamine dihydrochloride solution (CAS 524-36-7, SIGMA-ALDRICH P9380). (**c**, **d**) Show the supernatant sample extract from HmpT protein precipitate by methanol/water (1:1 v/v) and sonication treatment. All the samples were analyzed by Q Exactive mass spectrometer (Thermo Fisher Scientific, San Jose, CA, USA) coupled with UPLC. The MS spectra and MS/MS spectra were acquired with resolution of 70 000 and 17 500, respectively.

To answer these questions, HmpT was prepared and isolated in its whole-cell environment. From this preparation, the substrate of HmpT was extracted and subjected to mass spectrometric analysis. The complex of HmpT for pyridoxamine is detected by the high-resolution mass spectra in Figure 3. The mass spectra show that the substrate extracted from HmpT (Figure 3c) matches exactly the masses for standard pyridoxamine (Figure 3a). Further MS/MS analysis of the Pyridoxamine+H peak also shows similar profiles for both the standard and the substrate sample extracted from HmpT (Figures 2b and d). Taken together, these results suggest pyridoxamine as the natural substrate for HmpT in a native-like, biologically relevant environment.

### Pyridoxamine generally shows stronger interactions with the S-component

To further explore and compare the interactions of other possible substrates for HmpT, we investigated how these substrates (hydroxymethyl pyrimidine and the different active forms of vitamin B6: pyridoxine, pyridoxal and pyridoxamine) interact with the highly conserved residues in the EcfS-homologs indicated in Wang *et al*. [11]. Atomistic MD simulations were employed to see how these substrates interact with Glu41, His84, Gln87, Tyr120, Asn140 and Gln143. Supplementary Figures S1 and S2 show the root mean square deviation (RMSD) from the initial conformation of HmpT simulated in the ‘closed’ and ‘open’ conformation, respectively. From these figures, it can be seen that, in the first few nanoseconds of simulation, there was a sharp rise in the RMSD but goes on to fluctuate around the same RMSD value afterward, indicating that the global structure of the S-component is already quite stable up to ~60–80 ns. The attainment of stability within a short period of time is not unexpected for simulations where there are no explicit solvent molecules interacting with the transmembrane protein. Supplementary Figure S3 shows the time trajectory of the interaction energies of the six conserved residues with the different forms of vitamin B6 and hydroxymethyl pyrimidine for the system where HmpT was simulated in the open conformation. As can be seen from the figure, pyridoxamine interacts most strongly with most of the six residues (Glu 41, His 84 and Gln 143) compared with the other vitamin B6 species. Although hydroxymethyl pyrimidine does interact strongly with one of the conserved residues (Asn 140), overall, it is still pyridoxamine that can interact most strongly with residues in the binding pocket. Supplementary Figure S4, meanwhile, displays the energies of interaction of the different ligands with the conserved residues in the system where HmpT is simulated in the closed conformation. As with HmpT simulated in the open conformation, Supplementary Figure S4 also reveals that pyridoxamine has strong interactions with most of the conserved residues (His 84, Tyr 120 and Asn 140).

**Figure 4:**
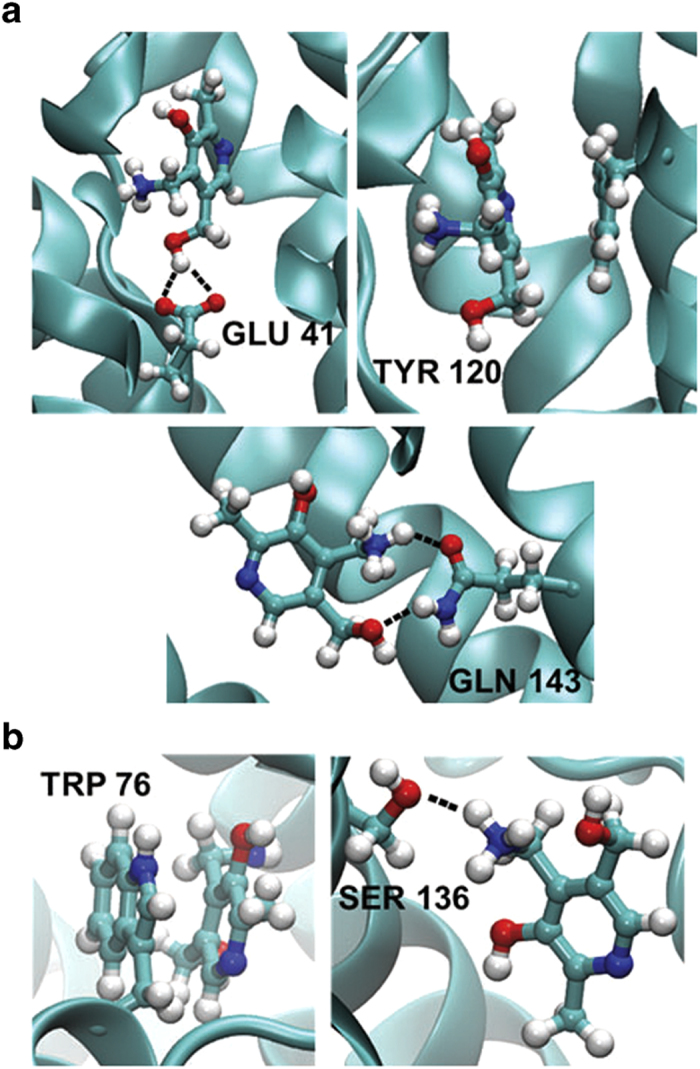
Snapshots of interactions between pyridoxamine and selected residues in the binding pocket. (**a**) Represents close-up snapshots of pyridoxamine interacting with the conserved residues Glu 41, Tyr 120 and Gln 143. Similar snapshots of pyridoxamine interacting with Trp 76 and Ser 136 are displayed in (**b**).

Figure 4 illustrates snapshots of the simulation of pyridoxamine binding with the S-component, highlighting the possible interactions that were observed. Figure 4a shows a close-up view of pyridoxamine interacting with the conserved residues Glu 41, Tyr 120 and Gln 143. As can be seen in this panel, it is possible that Glu 41 hydrogen bonds with one of the hydroxyl oxygens of pyridoxamine, while Gln 143 can hydrogen bond not only with the hydroxyl hydrogens but also with the amino hydrogens of pyridoxamine. Meanwhile, π-π interactions can also be seen to form between the aromatic ring of pyridoxamine and Tyr 120. Snapshots of other residues that were seen to also have strong interactions with pyridoxamine are illustrated in Figure 4b. Similar to Tyr 120, Trp 76 can also form π-π interactions with the aromatic ring of pyridoxamine, while the hydroxyl oxygen of Ser 136 can be seen to hydrogen bond with the amino hydrogens of pyridoxamine.

The orientational changes of the six conserved residues were also examined during the course of their interaction with pyridoxamine. In particular, the changes in the rotameric states of the side chain dihedrals of the six residues were observed. To describe the rotameric state, two dihedral angles were characterized: χ_1_ refers to the dihedral angle formed by the atoms N–C_α_–C_β_–C (where N is the backbone nitrogen atom); χ_2_ refers to the dihedral angle formed by the atoms C_α_–C_β_–C_γ_–C_δ_. The time trajectory of these dihedral angles together with the interaction energy between the pyridoxamine and a residue is presented in Supplementary Figure S5. Changes in the interaction energy between the ligand and a particular residue that correlates with changes in the rotameric state of the side chain of the same residue give an indication that the ligand and residue interacts in a particular manner that is affected by the orientation of the residue side chain. From Supplementary Figure S5, correlations can be seen between changes in the interaction energy and changes in the side chain dihedrals for Glu41 and Gln143. This is not unexpected if the hydrogen bonding interactions between pyridoxamine and these residues are important , as changes in the side chain dihedral angles can cause these hydrogen bonds to break.

### Loops 1 and 5 of the S-component have a role in the gating of the binding pocket opening

**Figure 5:**
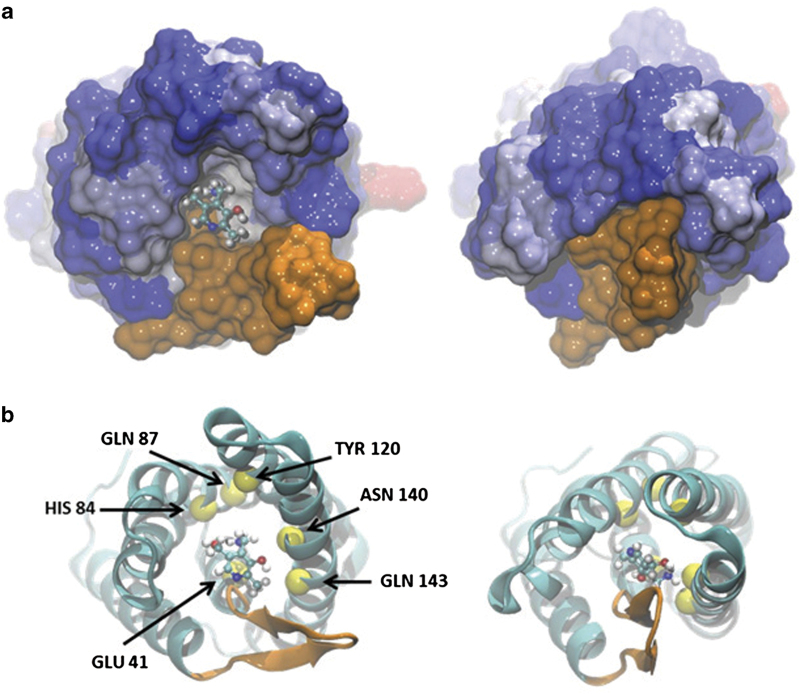
The closing of the gate in the presence of ligand as seen through the opening of the S-component. The S-component is drawn in surface rendering (**a**) and in cartoon rendering (**b**). The region colored orange in (**a**) represents Loop 1. The right side of (**b**) shows loop 1 closing over the opening of the binding pocket.

Loops SL1, SL3 and SL5 of the S-component have been previously speculated to have a role in substrate binding [20]. These are the loops that are found surrounding the opening of the binding pocket and join helices SH1-SH2, SH3-SH4 and SH5-SH6, respectively. In Supplementary Movies S1, S2, S3, it can be seen that some of these loops can behave as gates or lids that can cover the opening of the pocket. A portion of the simulation of HmpT in the closed conformation is visualized in Supplementary Movie S1. Loop SL5, together with helices SH5 and SH6, can be observed to slightly close over the binding pocket in the presence of the substrate. This is in good agreement with the role for loop SL5 that was reported for the S-component, RibU [25]. Interestingly, for the simulation of HmpT in the open position (Supplementary Movie S2), the gating role was identified to be played by loop SL1. This gating role for loop SL1 was also observed for the S-component ThiT [26]. Supplementary Movie S3 shows a portion of the HmpT simulation with no ligand. From this movie, a gating action was not observed from any of the loops within the same time period as shown in Supplementary Movies S1 and S2. Figure 5 shows how the gating occurs in that the opening can be observed to have closed considerably with the ligand no longer visible and with loop SL1 completely occluding the opening. Simulations also reveal that the closure of the opening can occur relatively quickly, within the first 2  ns of the simulation, when the ligand is present in the pocket.

### Mutant proteins exert an effect on pyridoxamine behavior in the binding pocket

Consistent with the speculation that the conserved residues of HmpT are necessary for protein function, mutations caused protein expression to drop markedly. This precluded further biophysical characterization of the effects of these mutated residues. In consonance with this experimental approach, the importance of the conserved residues was further explored by simulating HmpT where all the conserved residues were mutated into alanines. Likewise, the role of loops SL1 and SL5 were also explored further by simulating HmpT without those loops.

**Figure 6:**
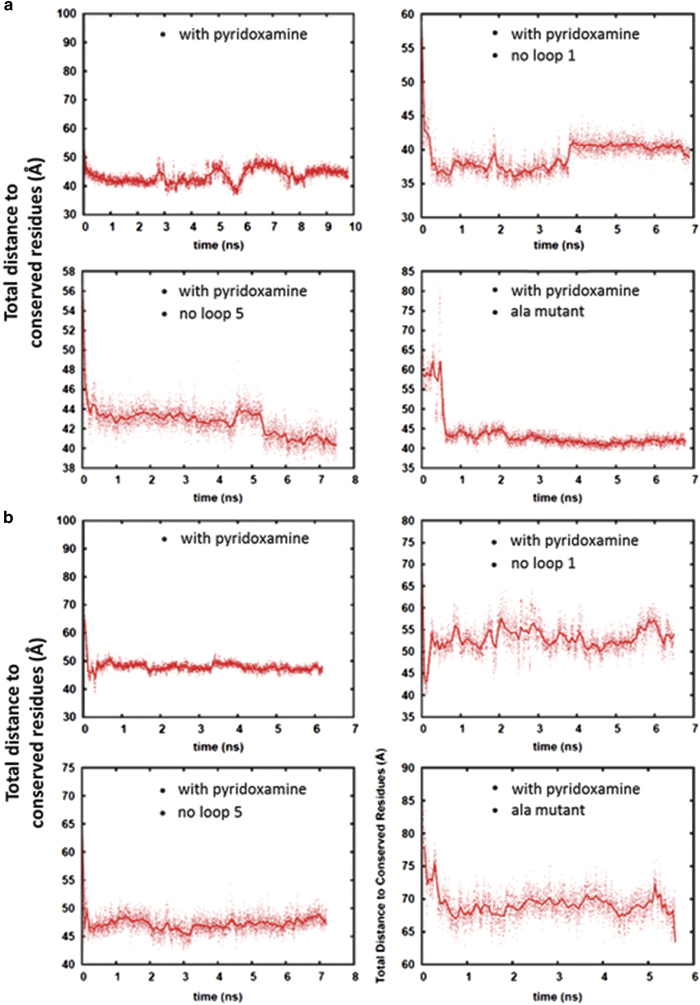
Total distance of the center of mass of pyridoxamine to the centers of mass of the side chains of the conserved residues. (**a**) Represents data from the simulation of HmpT in the closed conformation, while (**b**) represents data from the open conformation. The labels inside the boxes show the type of mutation that was imposed on HmpT.

Figure 6 presents the time trajectory of a ‘total distance from conserved residues’. This ‘total distance’ is calculated by measuring the distance between the center of mass of pyridoxamine to each of the six conserved residues and adding these distances together. From Figure 6a, it can be seen that the absence of loop SL1 from HmpT simulated in the closed conformation shows significant effect on how far the ligand is from the conserved residues. In contrast, the absence of loop SL5 appears to have no considerable effect on the total distance. But, for both of these mutants, even with the absence of the gating loops, the ligand is still able to interact with the conserved residues and able to maintain a constant total distance from them later on in the simulation. This indicates that once pyridoxamine is able to interact with the conserved residues, the loops have a minimal role in maintaining this interaction. We can also speculate that, with the presence of explicit water molecules, the role of the loops might become more important such that they may have a role in ensuring that water molecules are kept out of the binding pocket. The mutation of the conserved residues to alanines, likewise, does not show a marked effect on how freely the ligand can move in the binding pocket. Although it can be observed that in the first few nanoseconds of simulations, the ligand does not go near to the mutated residues as it did for the non-mutated HmpT. This could be due to the possible roles that the loops can also have such that, even in the absence of the conserved residues, the ligand is still able to stay within the binding pocket. Also, as was shown previously, pyridoxamine can also interact with residues in the binding pocket that are not among the conserved residues. The similar total distance measure for the simulation of HmpT in the open conformation is presented in Figure 6b. However, in the Ala mutant, it can be seen that though the ligand still tends to stay within a certain distance from the mutated residues, the total distance is farther away compared to the system with the non-mutated HmpT.

Taken together, the substrate for the S-component, HmpT, is identified to be pyridoxamine via mass spectrometric analyses. Mutations of the conserved residues in HmpT decreased protein expression markedly, lending support to their importance. Therefore, further investigation of the interactions of pyridoxamine with HmpT using MD simulations was carried out. The results reveal that pyridoxamine interacts strongly with most of the residues conserved among different S-components and that mutations of these residues to alanines exert an effect on the behavior of pyridoxamine in the HmpT binding pocket. In addition, MD simulations suggest that the closing of the binding pocket in the presence of the substrate is a relatively quick process and that loop SL1 has a major role in gating the binding pocket.

## Materials and Methods

### MD simulation

The Langevin dynamics simulations of the various EcfS systems were performed using the Chemistry at Harvard Molecular Mechanics (CHARMM) software package [27]. The fully atomistic models of the HmpT-ligand systems were simulated in a generalized Born implicit membrane model [28], which implicitly approximates a membrane core with a rigid, low-dielectric slab. For this work, the thickness of the membrane slab was set to 30 Å with the system temperature maintained at 303 K. The EcfS system was simulated in two orientations: the ‘closed’ conformation detailed by Wang *et al*. [11] that shows a ‘toppled’ S-component where the opening of the binding pocket faces the interior of the membrane, and the ‘open’ conformation where the EcfS protein was oriented such that the principal axis of the S-component is relatively parallel to the *z*-axis and the opening of the binding pocket faces the outside of the membrane. The CHARMM36 protein force field [29] was utilized with CMAP dihedral corrections [30] together with the CHARMM General Force Field for small molecules [31]. Four ligands are simulated with HmpT: hydroxymethyl pyrimidine (will be referred to as hmp), pyridoxine (pdx), pyridoxal (pdxl) and pyridoxamine (pdxm). Figure 2b draws out the structures of these ligands together with a snapshot (Figure 2a) of the simulation of HmpT in the open orientation with pyridoxamine as substrate. These ligands were placed near the opening of the S-component with the plane of the ligand molecules approximately parallel to the plane of the opening.

### Clones and protein preparation

The EcfS gene (GI:116100053) of HmpT was amplified from the Lactobacillus brevis ATCC 367 genomic DNA by polymerase chain reaction (PCR) and cloned into the expression vectors pET15b (Novagen, Madison, WI, USA). The N-terminus of HmpT was fused to a dodecahistidine affinity tag to facilitate the purification. The resulting clones were verified by DNA sequencing. The expression vector was transformed into *E. coli* strain C43(DE3) cells (Avidis, Saint Beauzire, France). Cells were grown to an optical cell density at 600 nm (OD600) of 1.2–1.5 at 37 °C, and the expression of recombinant proteins was induced by 0.25 mM isopropyl-β-D-thiogalactopyranoside (IPTG) for 3 h. Cells were harvested by centrifugation, resuspended in 25 mM Tris-HCl (pH 8.0), 150 mM NaCl and disrupted by sonication. Cell debris was removed by low-speed centrifugation for 10 min, and cell membranes were pelleted by ultracentrifugation at 100 000 *g* for 1 h. The membrane fraction was solubilized in 25 mM Tris-HCl (pH 8.0), 150 mM NaCl, 1% (w/v) n-dodecyl-β-D-maltopyranoside (DDM, Anatrace, Maumee, OH, USA) for 1.5 h at 4 °C. After another ultracentrifugation step at 100 000 *g* for 30 min, the supernatant was collected and loaded onto Ni2+-nitrilotriacetate affinity resin (Ni-NTA, Qiagen, Hilden, Germany) and washed with 25 mM Tris-HCl (pH 8.0), 150 mM NaCl, 20 mM imidazole and 0.02% DDM. The bound protein was eluted from the affinity resin with 25 mM Tris-HCl (pH 8.0), 150 mM NaCl, 250 mM imidazole and 0.02% DDM. The HmpT protein was concentrated to about 10 mg ml^−1^ before further purification by gel filtration (Superdex-200 10/30, GE Healthcare, Piscataway, NJ, USA). The buffer for gel filtration contained 25 mM Tris-HCl (pH 8.0), 150 mM NaCl and 0.32% (w/v) n-Nonyl-β-D-maltopyranoside (NM, Anatrace). The peak fractions were collected for further analysis.

### Mass spectrometry analysis

Methanol/water (1:1 v/v) was added to protein complex precipitate to extract the substrate and the mixture was sonicated for 20 min. After centrifugation, the supernatant was transferred to a new sample vial for mass spectrometry analysis. The samples were analyzed by Q Exactive mass spectrometer (Thermo Fisher Scientific) coupled with UPLC. A Thermo Hypersil Gold reverse phase column was used for LC separation with water and acetonitrile as mobile phases. Data acquisition was performed with positive ion mode and the mass range was within *m*/*z* 120–750. The MS spectra and MS/MS spectra were acquired with resolution of 70 000 and 17 500, respectively. The standard compound was analyzed under the same condition.
