# Supplementary material for: Pyridoxamine is a substrate of the energy-coupling factor transporter HmpT
Source: Cell Discov. 2015 Jul 14;1:15014–. doi: 10.1038/celldisc.2015.14 (PMC4860826; doi:10.1038/celldisc.2015.14)
Supplement: Supplementary Information [file celldisc201514-s1.doc]

Supplementary Information

# Pyridoxamine is a substrate of the energy-coupling factor transporter HmpT

Tingliang Wang^1,2§^, Armando Jerome de Jesus^3§^, Yigong Shi^1,2^* and Hang Yin^3,4^*

*^1^Ministry of Education Key Laboratory of Protein Science, Tsinghua University, Beijing 100084, China;*

*^2^Tsinghua-Peking Joint Center for Life Sciences, Center for Structural Biology, School of Life Sciences and School of Medicine, Tsinghua University, Beijing 100084, China;*

*^3^Department of Chemistry & Biochemistry, the BioFrontiers Institute, University of Colorado Boulder, Boulder, CO 80309-0596, USA*

*^4^Center of Basic Molecular Science, Department of Chemistry, Tsinghua University, Beijing 100084, China*

**List of Supplementary Materials**

| **Fig. S1.** Root mean square deviation of the HmpT model systems from their initial configuration. The HmpT was simulated in the closed conformation |  |
| --- | --- |
| **Fig. S2.** Root mean square deviation of the HmpT model systems from their initial configuration. The HmpT was simulated in the open conformation |  |
| **Fig. S3.** Energies of interaction between the different ligands and the six conserved residues in the binding pocket of the S-component that was simulated in the open conformation. |  |
| **Fig. S4.** Energies of interaction between the different ligands and the six conserved residues in the binding pocket of the S-component that was simulated in the closed conformation. |  |
| **Fig. S5.** The correlation between the interaction (red curve) and changes in side chain dihedral angles (orange and green curves) for some of the conserved residues for the simulation of HmpT in the closed conformation. |  |
| **Movie S6.** Movie showing the gating of loop SL5 for the HmpT system simulated in the closed conformation |  |
| **Movie S7.** Movie showing the gating of loop SL1 for the HmpT system simulated in the open conformation |  |
| **Movie S8.** Movie showing the absence gating for and empty HmpT system simulated in the closed conformation |  |
