# Supplementary material for: Pyridoxamine is a substrate of the energy-coupling factor transporter HmpT
Source: Cell Discov. 2015 Jul 14;1:15014–. doi: 10.1038/celldisc.2015.14 (PMC4860826; doi:10.1038/celldisc.2015.14)
Supplement: Supplementary Figure S2 [file celldisc201514-s3.doc]

**Wang et al. Supplementary Information Figure S2**

**
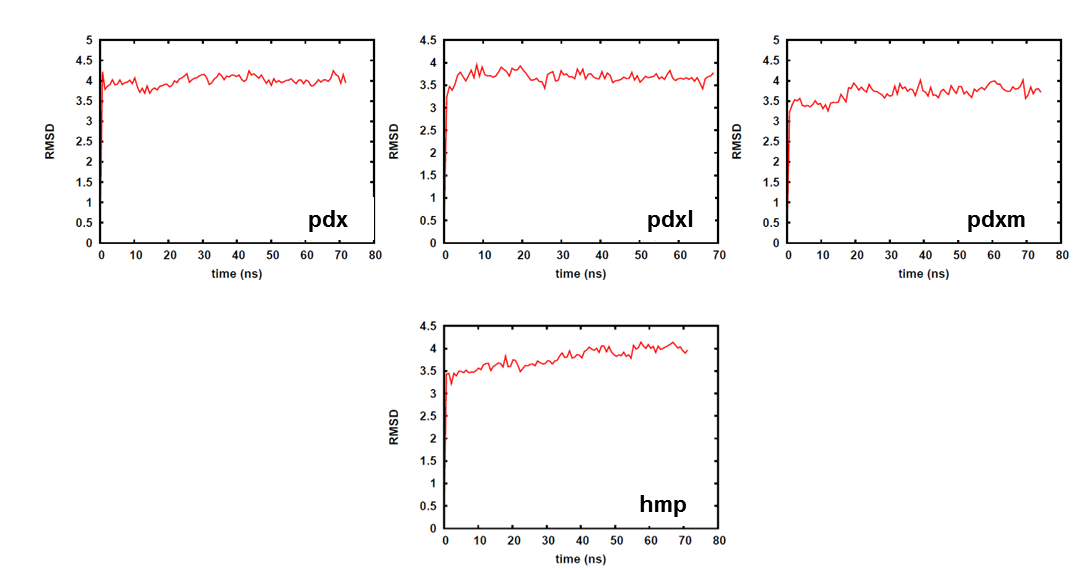
**

**Figure S2.** Root mean square deviation of the HmpT model systems from their initial configuration. The HmpT was simulated in the open conformation. After an initial increase, the RMSD levels off indicating the structure conformation has stabilized.
