# Supplementary material for: Pyridoxamine is a substrate of the energy-coupling factor transporter HmpT
Source: Cell Discov. 2015 Jul 14;1:15014–. doi: 10.1038/celldisc.2015.14 (PMC4860826; doi:10.1038/celldisc.2015.14)
Supplement: Supplementary Figure S4 [file celldisc201514-s5.doc]

**Wang et al. Supplementary Information Figure S4**

**
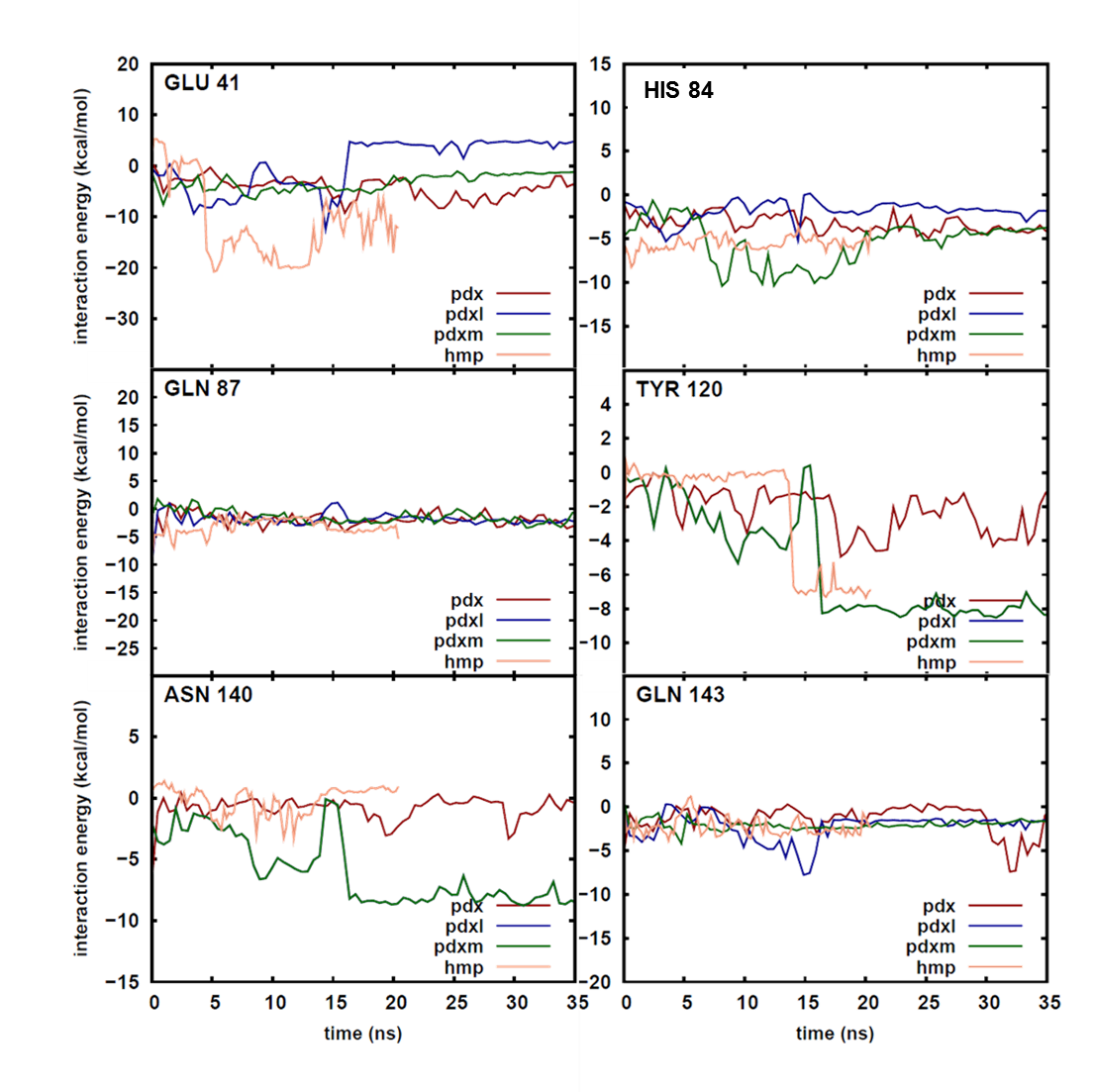
**

**Figure S4.** Calculated energies of interaction between the different ligands and the six conserved residues in the binding pocket of the S-component that was simulated in the closed conformation.
