# Supplementary material for: Pyridoxamine is a substrate of the energy-coupling factor transporter HmpT
Source: Cell Discov. 2015 Jul 14;1:15014–. doi: 10.1038/celldisc.2015.14 (PMC4860826; doi:10.1038/celldisc.2015.14)
Supplement: Supplementary Figure S5 [file celldisc201514-s6.doc]

**Wang et al. Supplementary Information Figure S5**

**
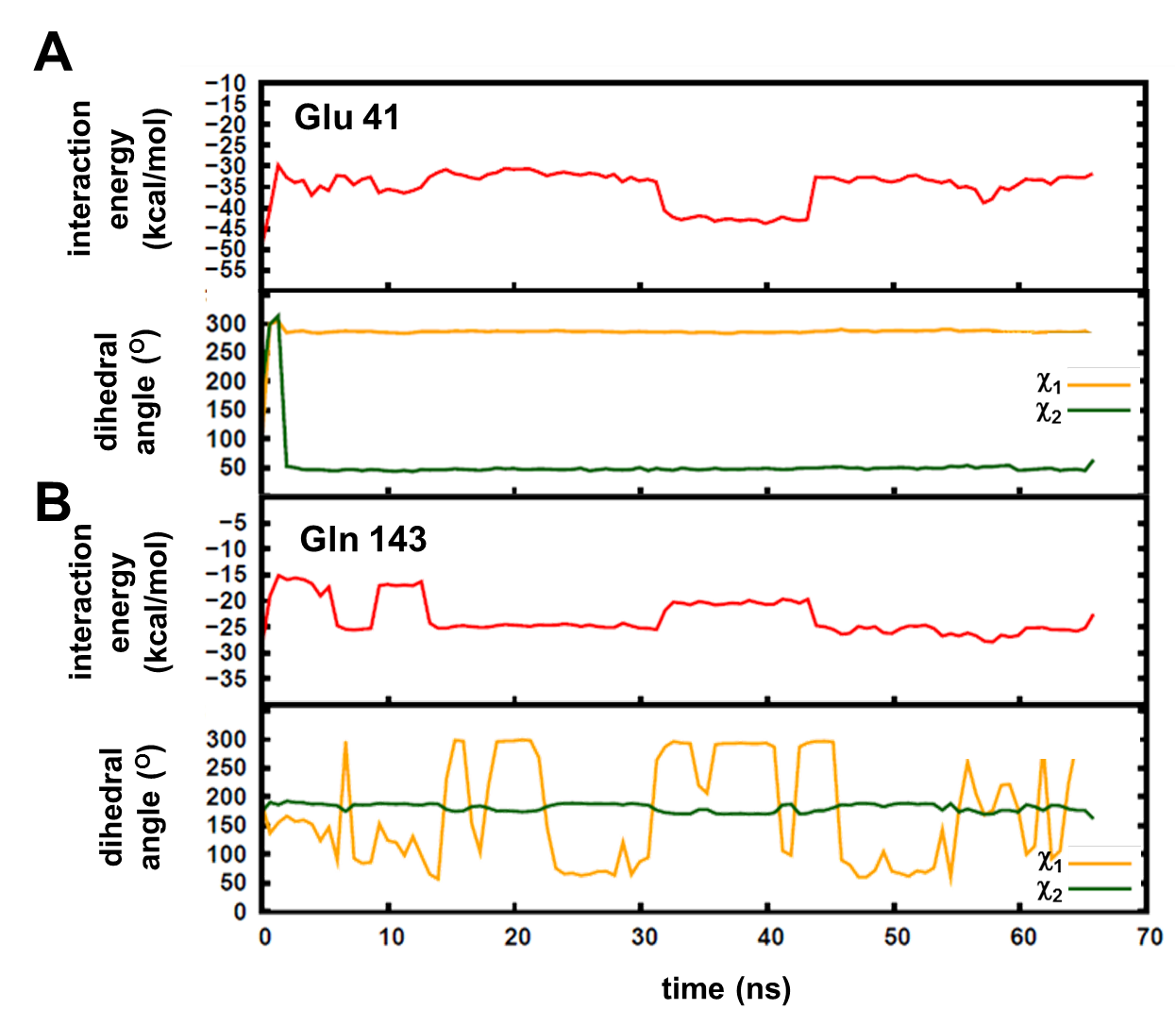
**

**Figure S5.** The correlation between the interaction (red curve) and changes in side chain dihedral angles (orange and green curves) for some of the conserved residues for the simulation of HmpT in the closed conformation
